# PancreaSeq Genomic Classifier (PancreaSeq GC) Improves Pancreatic Cyst Classification and Detection of Advanced Neoplasia: A Multi-institutional Validation Study

**DOI:** 10.1245/s10434-025-18848-8

**Published:** 2025-12-12

**Authors:** Aatur D. Singhi, Abigail I. Wald, Katelyn Smith, Lynn Wolkenstein, Robert D. Bubar, Ricardo Diaz-Aragon, Maria Grupillo, Danielle Guckin, Yi-Tak Lai, Randall E. Brand, Kevin McGrath, Walter G. Park, Patrick R. Pfau, Patricio M. Polanco, Nisa Kubiliun, John DeWitt, Jeffrey J. Easler, Aamir Dam, Shaffer R. Mok, Michael B. Wallace, Brian A. Boone, Wallis Marsh, Shyam Thakkar, Kimberly J. Fairley, Elham Afghani, Yasser Bhat, Sanjay Ramrakhiani, John Nasr, Nikhil R. Thiruvengadam, Asif Khalid, Kenneth Fasanella, Jennifer Chennat, Rohit Das, Harkirat Singh, Adam Slivka, Charles Gabbert, Tarek Sawas, Thomas Tielleman, Hendrikus Dutch Vanderveldt, Anna Tavakkoli, Lynette M. Smith, Anju H. Singhi, Sara A. Singhi, Stephanie Romutis, Sultan Mahmood, Amy E. Hosmer, Julia McNabb-Baltar, Anne Marie Lennon, Ralph H. Hruban, Nuha Shaker, Alessandro Paniccia, Amer Zureikat, Kenneth K. Lee, Melanie Ongchin, Herbert Zeh, Rebecca Minter, C. Max Schmidt, Jin He, Marina N. Nikiforova

**Affiliations:** 1 Department of Pathology, University of Pittsburgh Medical Center, Pittsburgh, PA USA; 2 Department of Medicine, University of Pittsburgh Medical Center, Pittsburgh, PA USA; 3 Department of Medicine, Stanford University, Stanford, CA USA; 4 Department of Medicine, University of Wisconsin, Madison, WI USA; 5 Department of Surgery, University of Texas Southwestern Medical Center, Dallas, TX USA; 6 Department of Medicine, University of Texas Southwestern Medical Center, Dallas, TX USA; 7 Department of Gastroenterology and Hepatology, Indiana University Health Medical Center, Indianapolis, IN USA; 8 Piedmont Advanced Gastroenterology Atlanta, Atlanta, GA USA; 9 Department of Gastrointestinal Oncology, Moffitt Cancer Center, Tampa, FL USA; 10 Department of Medicine, Division of Gastroenterology and Hepatology, Mayo Clinic, Jacksonville, FL USA; 11 Department of Surgery, West Virginia University Health Sciences Center, Morgantown, WV USA; 12 Department of Medicine, Section of Gastroenterology & Hepatology, West Virginia University Health Sciences Center, Morgantown, WV USA; 13 The Sol Goldman Pancreatic Cancer Research Center, Department of Medicine, Johns Hopkins Medical Institutions, Baltimore, MD USA; 14 Department of Gastroenterology, Palo Alto Medical Foundation (PAMF), Mountain View, CA USA; 15 Department of Medicine, Wheeling Hospital, West Virginia University Health Sciences Center, Morgantown, WV USA; 16 Department of Medicine, Division of Gastroenterology and Hepatology, Loma Linda University Medical Center, Loma Linda, CA USA; 17 Department of Biostatistics, College of Public Health, University of Nebraska Medical Center, Omaha, NE USA; 18 The Sol Goldman Pancreatic Cancer Research Center, Department of Pathology, Johns Hopkins Medical Institutions, Baltimore, MD USA; 19 Department of Surgery, University of Pittsburgh Medical Center, Pittsburgh, PA USA; 20 Department of Clinical Sciences, Surgery, University of Texas Southwestern, Dallas, TX USA; 21 Department of Surgery, University of Wisconsin, Madison, WI USA; 22 Department of Surgery, Indiana University Health Medical Center, Indianapolis, IN USA; 23 The Sol Goldman Pancreatic Cancer Research Center, Department of Surgery, Johns Hopkins Medical Institutions, Baltimore, MD USA

**Keywords:** Intraductal papillary mucinous neoplasm, Mucinous cystic neoplasm, Intraductal oncocytic papillary neoplasm, Serous cystadenoma, Pseudocyst, Pancreatic neuroendocrine tumor, Pancreatic ductal adenocarcinoma

## Abstract

**Background:**

The preoperative classification of pancreatic cysts and detection of advanced neoplasia (high-grade dysplasia/pancreatic ductal adenocarcinoma [PDAC]) represents a significant diagnostic challenge. A prospective, multi-institutional study found that DNA-based testing (PancreaSeq) of pancreatic cyst fluid (PCF) improved the assessment of pancreatic cysts. Notable imitations to PancreaSeq necessitated the development of a DNA/RNA-based panel called PancreaSeq Genomic Classifier (GC). We validated PancreaSeq GC on a prospective patient cohort.

**Patients and Methods:**

PancreaSeq GC was blindly tested on a prospective cohort of 241 patients with diagnostic follow-up. The performance of PancreaSeq GC in this cohort, which included 186 mucinous cysts (97 with advanced neoplasia), was benchmarked against traditional diagnostics and its DNA-only predecessor, PancreaSeq.

**Results:**

PancreaSeq GC achieved 94.6% sensitivity and 96.4% specificity (area under the curve [AUC] of 0.955) for mucinous cysts. In comparison, increased fluid viscosity, elevated carcinoembryonic antigen (CEA), and PancreaSeq testing had lower sensitivities (71.4–88.2%) and lower AUC (0.824–0.941); however, PancreaSeq specificity was 100%. McNemar’s test demonstrated higher sensitivity of PancreaSeq GC versus PancreaSeq for mucinous cysts (*p* < 0.001). For advanced neoplasia, PancreaSeq GC had 86.6% sensitivity and 97.9% specificity (AUC of 0.923), with McNemar’s test confirming improved sensitivity over PancreaSeq (*p* = 0.031). Worrisome features, malignant cytopathology, and high-risk stigmata had lower sensitivities (44.3–84.5%) and lower AUC (0.604–0.892), while PancreaSeq had identical specificity to PancreaSeq GC. PancreaSeq GC had high accuracy in classifying intraductal oncocytic papillary neoplasms (IOPNs), intraductal tubulopapillary neoplasms (ITPNs), and cystic pancreatic neuroendocrine tumors (cPanNETs), with 97.1–100% sensitivity and 100% specificity.

**Conclusions:**

This validation study demonstrates statistically significant improvements in PancreaSeq GC for mucinous cysts and advanced neoplasia, establishing it as a clinically valuable tool for the preoperative evaluation of pancreatic cysts.

**Supplementary Information:**

The online version contains supplementary material available at 10.1245/s10434-025-18848-8.

Owing to the widespread use of cross-sectional imaging, pancreatic cysts are increasingly encountered and have become a significant source of anxiety for both physicians and patients alike. These lesions encompass a heterogeneous group, including non-neoplastic cysts, benign cystic neoplasms, and cystic neoplasms with varying potential for malignant transformation.^1^ Mucinous cysts, such as intraductal papillary mucinous neoplasms (IPMNs), intraductal oncocytic papillary neoplasms (IOPNs), intraductal tubulopapillary neoplasms (ITPNs), and mucinous cystic neoplasms (MCNs), are precursors to pancreatic ductal adenocarcinoma (PDAC), but only a small subset will undergo malignant transformation in a patient’s lifetime. In contrast, nonmucinous cysts, such as pseudocysts and serous cystadenomas (SCAs), are benign lesions with essentially no malignant potential.^2^ Therefore, the accurate classification of pancreatic cysts into mucinous versus nonmucinous types, and further stratification based on the presence versus absence of advanced neoplasia (high-grade dysplasia/microscopic PDAC), is critical for guiding patient surveillance and surgical management.

Traditionally, diagnostic approaches for pancreatic cysts have relied on the combination of clinical presentation, imaging characteristics, and cyst fluid analysis, such as carcinoembryonic antigen (CEA) levels and cytopathologic evaluation.^3–6^ However, these methods have demonstrated limited sensitivity, specificity, and overall accuracy, particularly in the setting of an indeterminate cyst or the detection of advanced neoplasia.^7^ Molecular testing of pancreatic cyst fluid has emerged as a powerful adjunct to existing diagnostic modalities, offering the potential to improve diagnostic accuracy and inform more personalized management strategies.^8^ In a prospective, observational study, Paniccia et al*.* evaluated the performance of a 22-gene DNA-based next-generation sequencing panel, PancreaSeq, on over 1800 pancreatic cyst specimens from 31 centers across the USA. The authors demonstrated that PancreaSeq significantly outperformed traditional approaches, achieving 90% sensitivity and 100% specificity for mucinous cysts and 88% sensitivity and 98% specificity for advanced neoplasia.^9^ As a result, the International Evidence-Based Kyoto Guidelines for the Management of IPMNs of the Pancreas highlighted the clinical utility of DNA-based next-generation sequencing (NGS) for pancreatic cyst classification and detection of advanced neoplasia.^10^

Recently, we reported the development and validation of PancreaSeq Genomic Classifier (GC), a combined DNA- and RNA-based NGS platform that targets 74 genes to evaluate five classes of genomic alterations, designed to improve the diagnostic accuracy of pancreatic cyst classification and risk stratification.^11^ PancreaSeq Genomic Classifier (GC) builds upon earlier versions of PancreaSeq by incorporating DNA point and indel mutations, chromosomal alterations, RNA gene expression changes, and fusion gene transcripts.^12–17^ However, to date, PancreaSeq GC has not been validated on a prospectively collected cohort of endoscopic ultrasound-guided fine-needle aspiration (EUS-FNA)-obtained pancreatic cyst fluid specimens. The aims of this study were to (1) determine the diagnostic performance of PancreaSeq GC using the multi-institutional cohort reported by Paniccia et al*.*, (2) compare PancreaSeq GC with traditional diagnostic modalities and its predecessor, PancreaSeq, and (3) assess the clinical utility of incorporating additional genomic alterations associated with advanced neoplasia, specifically *CTNNB1* and *CDKN2A*.^7^

## Patients and Methods

### Study Design

A double-blind validation analysis was performed utilizing the prospectively collected multi-institutional patient cohort described by Paniccia et al*.*^9^ This cohort comprised 1856 pancreatic cyst specimens obtained via endoscopic ultrasound-guided fine-needle aspiration (EUS-FNA) from 31 medical centers across the USA between 2016 and 2020. A total of 246 patients within this cohort underwent surgical resection, permitting definitive histopathologic classification and enabling use of these patient cases as a reference standard for PancreaSeq GC validation. For each of these 246 resected patients, previously extracted and banked nucleic acids were retrieved for further testing. All samples had been previously analyzed using the original 22-gene DNA-based PancreaSeq panel. For this study, residual DNA and RNA from the same EUS-FNA specimens were reanalyzed using the PancreaSeq GC platform.

To maintain study objectivity and regulatory compliance, all specimens and associated clinical metadata were deidentified using an institutional review board (IRB)-approved honest broker system prior to PancreaSeq GC testing (IRB no. STUDY19070069). The honest broker system served as a neutral intermediary responsible for delinking clinicopathologic data and assigning anonymized study identifiers. As a result, all personnel performing and interpreting PancreaSeq GC testing were blinded to the clinical, radiologic, endoscopic, cytopathologic, laboratory, and histopathologic data. Upon completion of all PancreaSeq GC testing, diagnostic performance metrics were unblinded by the honest broker for statistical analysis using previously established histopathologic classifications as the reference standard.^18,19^ The primary objectives of this study were to assess the diagnostic sensitivity, specificity, negative predictive value (NPV), positive predictive value (PPV), and overall accuracy of PancreaSeq GC in classifying pancreatic cysts as mucinous versus nonmucinous and in detecting the presence versus absence of advanced neoplasia. Secondary objectives included comparative performance analysis against traditional diagnostic modalities: clinical presentation, fluid viscosity, CEA analysis, cytopathologic findings, worrisome features, high-risk stigmata, and PancreaSeq results. On the basis of the International Evidence-Based Kyoto Guidelines for the Management of IPMNs,^10^ worrisome features are defined as the presence of a cyst measuring ≥ 3.0 cm, a thickened or enhancing cyst wall, nonenhancing mural nodules, a main pancreatic duct diameter of 5–9 mm, an abrupt change in duct caliber with distal pancreatic atrophy, lymphadenopathy, elevated serum carbohydrate antigen (CA) 19-9, cyst growth exceeding 5 mm per year, or pancreatitis attributable to the cyst. High-risk stigmata are defined by the presence of obstructive jaundice in a patient with a cystic lesion in the head of the pancreas, an enhancing mural nodule ≥ 5 mm, a main pancreatic duct diameter ≥ 10 mm in the absence of other causes, and malignant cytopathology. Malignant cytopathology was defined as at least suspicious for adenocarcinoma.

### PancreaSeq GC Testing

**Fig. 1:**
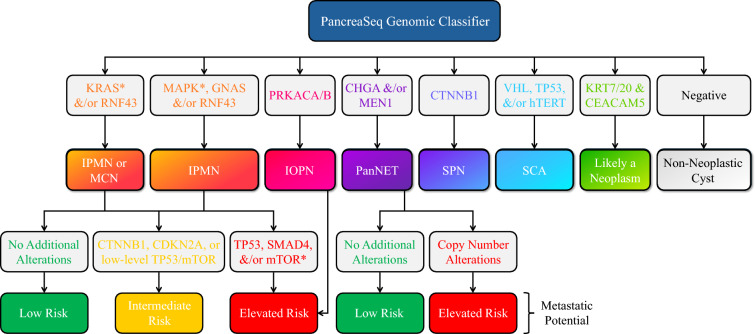
The PancreaSeq Genomic Classifier (PancreaSeq GC) uses an algorithmic approach to classify and risk stratify pancreatic cysts; PancreaSeq GC integrates targeted DNA- and RNA-based next-generation sequencing (NGS) of endoscopic ultrasound-guided fine-needle aspiration (EUS-FNA)-obtained pancreatic cyst fluid with a bioinformatic genomic classifier; the initial step of PancreaSeq GC is to determine pancreatic cyst type using five classes of genomic alterations; subsequent risk stratification incorporates additional alterations associated with advanced neoplasia or metastatic potential

PancreaSeq GC has been implemented as a clinically available laboratory-developed test, accessible to providers for guiding the evaluation of patients with pancreatic cysts. This molecular assay employs targeted DNA/RNA-based NGS of 74 genes encompassing five genomic alteration classes: (1) DNA point mutations and insertions/deletions, (2) chromosomal copy number alterations, (3) RNA gene expression profiles, (4) fusion gene transcripts, and (5) *CEACAM5* mRNA quantification by reverse transcription quantitative polymerase chain reaction (RT-qPCR).^11^ Extracted DNA and RNA were quantitated on the Glomax Discover using the QuantiFluor ONE dsDNA System and the QuantiFluor RNA system, respectively (Promega, Madison, WI, USA). NGS libraries (Supplementary Materials and Methods) were created from 10 ng of DNA and 10 ng of RNA using the Ion AmpliSeq Library kit PLUS and Ion Xpress Barcode Adapters as previously described. The libraries were normalized for template preparation on the Ion Chef and sequenced on an Ion S5 System according to the manufacturer’s instructions (ThermoFisher Scientific, Waltham, MA, USA). The Torrent Suite Software version 5.12 (Thermo Fisher Scientific) and an in-house-developed software Variant Explorer version 2 were used for data analysis and interpretation. Each variant was prioritized according to the 2017 Association for Molecular Pathology (AMP)/American Society of Clinical Oncology (ASCO)/College of American Pathologists (CAP) joint consensus guidelines for interpretation of sequence variants in cancer using a tier-based system.^20^ Tier I, tier II, and tier III variants were identified; however, only tier I and tier II variants were used for subsequent analysis. In parallel, *CEACAM5* testing was performed using TaqMan One-step RT to Ct Master Mix kit and run on the ABI7500 real-time PCR instrument according to manufacturer’s instructions (Applied Biosystems). Relative quantity of *CEACAM5* in gene expression units (GEU) was calculated according to the 2^−ΔΔCt^ method.^21^ Categorization of variants with specific diagnostic categories was performed as summarized in Fig. 1.

### Statistical Analysis

Sensitivity, specificity, NPV, PPV, and overall accuracy with 95% Wilson confidence intervals were calculated for each diagnostic parameter. The true positive fraction (sensitivity) and the false positive fraction (1-specificity) were plotted with receiver operating characteristic curves (Supplementary Materials and Methods). Area under the curve (AUC) was computed using the trapezoidal method by DeLong.^22^ All statistical analyses were performed using the SPSS Statistical software, version 29 (IBM, Armonk, NY, USA) and R software. Statistical significance was defined as a *p*-value of < 0.05. The primary comparisons between PancreaSeq GC, PancreaSeq, and traditional diagnostic modalities were prespecified on the basis of the study’s objectives. Therefore, formal adjustments for multiple comparisons were not performed, and *p*-values are presented to allow for direct interpretation of each planned comparison.

## Results

### PancreaSeq GC Testing Results

**Fig. 2:**
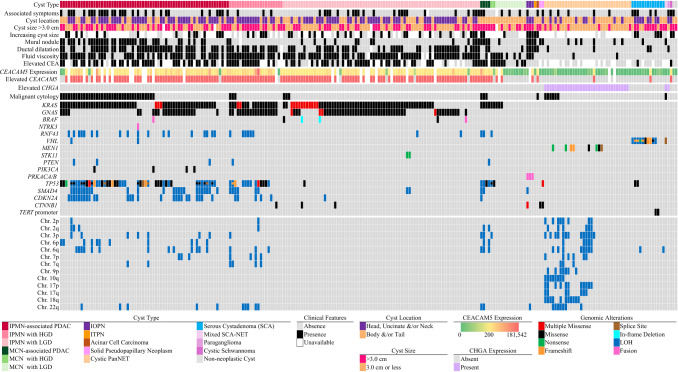
Oncoplot summarizing the clinical, pathologic, and genomic features of 241 pancreatic cysts; each column represents an individual patient case, grouped by cyst type (top); clinical annotations include symptoms, location, size, growth, mural nodule, ductal dilatation, fluid viscosity, and elevated CEA; *CEACAM5* (green–yellow–red gradient) and *CHGA* (purple) gene expression, malignant cytology (black), and DNA/RNA-based alterations are shown; genomic alterations (colored by type) are shown for recurrently altered genes, including *KRAS*, *GNAS*, *BRAF*, *RNF43*, *TP53*, *SMAD4*, and *CDKN2A*, along with chromosomal arm-level loss (blue)

Residual nucleic acids were sufficient for PancreaSeq GC testing in 241 of 246 (98%) EUS-FNA specimens (Supplementary Data and Supplementary Table 1), which included DNA/RNA-targeted next-generation sequencing (NGS) in 241 (98%) patient cases and *CEACAM5* testing in 215 (87%) patient cases (Fig. 2). DNA/RNA-targeted NGS was successful in all specimens that yielded evaluable *CEACAM5* results. This cohort corresponded to 241 surgically resected pancreatic cysts, which comprised 186 (77%) mucinous and 55 (23%) nonmucinous cysts. Among the 186 mucinous cysts, 97 (52%) harbored advanced neoplasia, with high-grade dysplasia alone identified in 26 (of 97, 27%) cases and microscopic pancreatic ductal adenocarcinoma (PDAC) in 71 (73%) cases.

PancreaSeq GC testing identified a wide spectrum of DNA- and RNA-based alterations. The most frequent DNA-based alterations involved genes within the mitogen-activated protein kinase (MAPK) and protein kinase A (PKA) signaling pathways. Among the MAPK/PKA pathway genes, *KRAS* mutations were the most prevalent (58%), followed by *GNAS* (37%), and *BRAF* (2%). Multiple *KRAS* and *GNAS* missense mutations were identified in 16 (7%) and 4 (2%) cases, respectively. Alterations in *KRAS*, *GNAS,* and *BRAF* were only identified in mucinous cysts, while multiple *KRAS* and/or *GNAS* missense mutations were specific to IPMNs. Other DNA-based alterations included *TP53* (28%), *VHL* (18%), *SMAD4* (15%), *CDKN2A* (14%), *RNF43* (12%), *MEN1* (3%), *CTNNB1* (3%), *PIK3CA* (2%), *PTEN* (2%), *STK11* (1%), and the hTERT promoter (1%). Most mutations in *TP53* (91%), *SMAD4* (100%), *CDKN2A* (100%), *RNF43* (93%), *CTNNB1* (67%), *PIK3CA* (100%), *PTEN* (100%), and *STK11* (100%) co-occurred with MAPK/PKA pathway alterations. The combination of MAPK/PKA and *TP53*, *SMAD4*, *CDKN2A*, *CTNNB1*, *PIK3CA*, and *PTEN* alterations correlated with the presence of advanced neoplasia in 98%, 94%, 97%, 33%, 100%, and 100% of cases, respectively. Mutations in *VHL* and *MEN1* were mutually exclusive of MAPK/PKA pathway alterations and corresponded to an SCA or cystic pancreatic neuroendocrine tumor (cPanNET). In addition to *VHL*, other alterations identified in SCAs included *TP53* and the *hTERT* promoter. Chromosomal abnormalities were detected in 64 of 241 (27%) specimens. The most prevalent chromosomal alteration occurred in the short arm of chromosome 6 (12%), followed by 3p (10%), 22q (8%), 6p (8%), and others. Multiple chromosomal abnormalities were identified in 43% of advanced neoplasia patient cases and 59% of cPanNETs. Of note, none of the low-grade mucinous cysts had chromosomal alterations.

RNA-based alterations encompassed changes in gene expression and fusion gene transcripts. Among 215 pancreatic cysts, *CEACAM5* expression ranged from 0 to 181,423 GEU (mean 9611 GEU; median, 2269 GEU) and was elevated (> 200 GEU) in 145 (67%) patient cases. ALL *CEACAM5*-elevated cases co-expressed *KRT7* and/or *KRT20*. *CEACAM5* overexpression was observed in 141 (of 162, 87%) mucinous cysts and 4 (of 53, 8%) nonmucinous cysts. The four nonmucinous cysts were a cystic acinar cell carcinoma (ACC), a cPanNET (with elevated *CHGA*), an SCA (with a *VHL* alteration), and a cystic schwannoma. *CHGA* overexpression was also identified and restricted to nonmucinous cysts and detected in 33 (of 34, 97%) cPanNETs, 1 (100%) mixed SCA-NET, and 1 (100%) peripancreatic paraganglioma. Detected fusion transcripts comprised *PRKACA/B* (*n* = 3), *BRAF* (*n* = 2), and *NTRK3* (*n* = 1) alterations. All *PRKACA/B* fusions were associated with an IOPN. In contrast, *BRAF* and *NTRK3* fusion genes correlated with an IPMN. Because of their association with IOPNs, *PRKACA/B* fusion genes corresponded to the presence of advanced neoplasia, but a similar correlation with advanced neoplasia was not observed with *NTRK3* and *BRAF* fusion genes.

### Diagnostic Performance and Comparison with Other Diagnostic Modalities

**Fig. 3:**
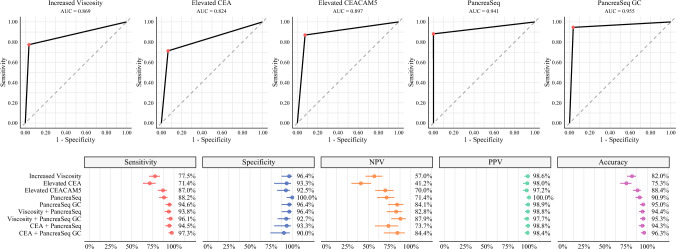
Diagnostic performance of individual and combined biomarkers in identifying mucinous pancreatic cysts; (top) receiver operating characteristic (ROC) curves for increased cyst fluid viscosity, elevated CEA, elevated *CEACAM5* expression, PancreaSeq, and PancreaSeq GC, with corresponding area under the curve (AUC) values; (bottom) forest plots summarizing sensitivity, specificity, negative predictive value (NPV), positive predictive value (PPV), and overall accuracy for each biomarker alone and in combination; red, blue, orange, green, and purple points represent sensitivity, specificity, NPV, PPV, and overall accuracy, respectively, with horizontal bars indicating 95% confidence intervals

For the identification of mucinous cysts, PancreaSeq GC demonstrated a sensitivity of 94.6%, a specificity of 96.4%, a negative predictive value (NPV) of 84.1%, a positive predictive value (PPV) of 98.9%, and an overall accuracy of 95% (Fig. 3 and Supplementary Fig. 1). In comparison, traditional assessments such as increased fluid viscosity and elevated cyst fluid CEA yielded lower sensitivity, NPV, PPV, and accuracy. Notably, increased fluid viscosity exhibited a specificity of 96.4%, comparable to that of PancreaSeq GC. McNemar’s test revealed a statistically significant difference in sensitivity, PPV, and accuracy between PancreaSeq GC and the other assays (*p* ≤ 0.013), but no difference in specificity and NPV (*p* ≥ 0.500) (Supplementary Fig. 2). Moreover, no difference in diagnostic performance was observed between PancreaSeq GC and the combination of increased fluid viscosity and elevated CEA, but data were available for only 166 (68%) patient cases. The addition of PancreaSeq GC to either fluid viscosity assessment or CEA testing improved the sensitivity, NPV, and accuracy beyond that of either modality alone. PancreaSeq GC alone provided the highest specificity and PPV among all tested approaches.

**Fig. 4:**
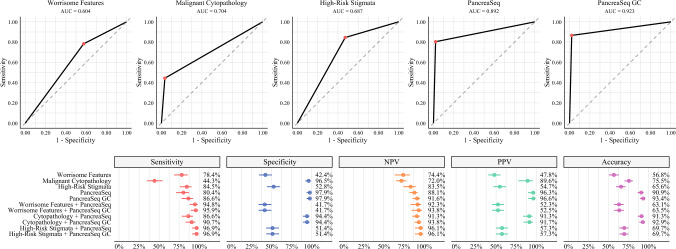
Diagnostic performance of clinical, imaging, cytologic, and genomic findings in detecting advanced neoplasia in mucinous cysts; (top) ROC curves for worrisome features, malignant cytopathology, high-risk stigmata, PancreaSeq, and PancreaSeq GC, with corresponding AUC values; (bottom) forest plots summarizing sensitivity, specificity, NPV, PPV, and overall accuracy for each criterion alone and in combination; red, blue, orange, green, and purple points represent sensitivity, specificity, NPV, PPV, and overall accuracy, respectively, with horizontal bars indicating 95% confidence intervals

PancreaSeq GC had a sensitivity, specificity, NPV, PPV, and overall accuracy for advanced neoplasia of 86.6%, 97.9%, 91.6%, 96.6%, and 93.4%, respectively (Fig. 4). Similar to mucinous cysts, PancreaSeq GC had high diagnostic performance in the detection of advanced neoplasia as compared with worrisome features, malignant cytopathology, and high-risk stigmata in sensitivity (44.3–84.5%), specificity (42.4–96.5%), NPV (72.0–83.5%), PPV (47.8–89.6%), and accuracy (56.8–75.5%). A statistically significant difference was observed for sensitivity and PPV between PancreaSeq GC and malignant cytopathology (*p* < 0.001), while specificity and NPV were statistically different between PancreaSeq GC and both worrisome features and high-risk stigmata (*p* < 0.001) (Supplementary Fig. 3). However, the overall accuracy of PancreaSeq GC for advanced neoplasia was significantly different between all three parameters (*p* < 0.001). In combination with PancreaSeq GC, the sensitivity, NPV, PPV, and accuracy for worrisome features, malignant cytopathology, and high-risk stigmata improved to 90.7–96.9%, 93.8–96.1%, 52.5–91.7%, 63.5–92.9%, respectively. The specificity of these diagnostic parameters decreased by 0.7–2.1% with the inclusion of PancreaSeq GC. Furthermore, the highest overall accuracy for advanced neoplasia was 93.4% and was achieved using PancreaSeq GC alone.

Among 12 SCAs, cytopathology was consistent with a serous neoplasm for one (8%) patient. In contrast, PancreaSeq GC had 83.3% sensitivity, 100% specificity, 99.1% NPV, 100% PPV, and 99.2% overall accuracy. The sensitivity of cytopathologic evaluation for cystic neuroendocrine-type neoplasms (cPanNETs, mixed SCA-NET, and cystic schwannoma) was only 16.7% but 100% specificity, 87.2% NPV, 100% PPV, and 87.6% accuracy. PancreaSeq GC yielded a sensitivity, specificity, NPV, PPV, and overall accuracy of 97.2%, 100%, 99.5%, 100%, and 99.6%, respectively. With regard to the presence of distant metastasis for cPanNETs, ≥ 3 chromosomal alterations in a cPanNET had 100% sensitivity, 64.3% specificity, 100% NPV, 23.1% PPV, and 67.7% accuracy (Supplementary Fig. 4). Among individual chromosomal alterations, loss of heterozygosity (LOH) at chromosome 10q was the only statistically significant alteration (100% versus 11%, *p* = 0.004) with a sensitivity of 100%, specificity of 89.3%, NPV of 100%, PPV of 50.0%, and accuracy of 90.3%. In comparison, a size of > 2.0 cm had a 66.7% sensitivity, 64.3% specificity, 94.7% NPV, 16.7% PPV, and 64.5% accuracy.

To summarize, for the primary goal of not missing high-grade dysplasia/microscopic PDAC, the most important metric is sensitivity. PancreaSeq GC demonstrated a statistically significant advantage, with a sensitivity of 86.6% for advanced neoplasia, outperforming traditional modalities (44.3–84.5%). Conversely, when contemplating surgery, the goal is often to be certain high-grade dysplasia/microscopic PDAC is present, making specificity paramount to avoid unnecessary operations. PancreaSeq GC exhibited high diagnostic performance in this domain, maintaining a high specificity of 97.9%. In essence, these results show that PancreaSeq GC improves upon traditional modalities, simultaneously enhancing the ability to confidently rule out advanced neoplasia, while also accurately confirming its presence, thereby addressing the two most important clinical challenges in pancreatic cyst assessment.

### Comparison of PancreaSeq GC and PancreaSeq for Mucinous Cysts and Advanced Neoplasia

McNemar’s test demonstrated statistically significant differences between PancreaSeq GC and PancreaSeq in the detection of mucinous cysts. PancreaSeq GC exhibited higher sensitivity (94.6% versus 88.2%, *p* < 0.001) and overall accuracy (95.0% versus 90.9%, *p* = 0.013); however, PancreaSeq had a higher PPV than PancreaSeq GC (100% versus 98.9%, *p* < 0.001). No variation in specificity was observed, and the NPV remained unchanged (*p* = 0.500). For advanced neoplasia, PancreaSeq GC showed improved sensitivity (86.6% versus 80.4%, *p* = 0.031), PPV (96.6% versus 96.3%, *p* = 0.031), and accuracy (93.4% versus 90.9%, *p* = 0.031) compared with PancreaSeq, but with no difference in specificity and NPV.

**Fig. 5:**
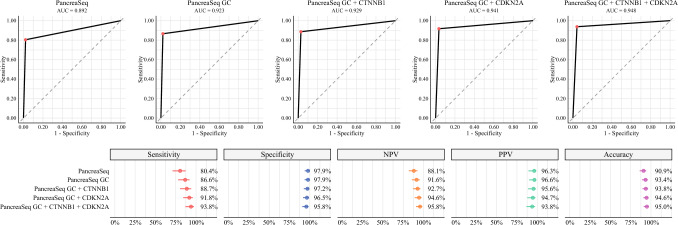
Diagnostic performance of genomic biomarker testing in identifying advanced neoplasia; (top) ROsC curves for PancreaSeq, PancreaSeq GC, and PancreaSeq GC combined with mutations in *CTNNB1* and/or *CDKN2A*, with corresponding AUC values; (bottom) forest plots summarizing sensitivity, specificity, NPV, PPV, and overall accuracy for each classifier; red, blue, orange, green, and purple points represent sensitivity, specificity, NPV, PPV, and overall accuracy, respectively, with horizontal bars indicating 95% confidence intervals

To evaluate the clinical utility of incorporating *CTNNB1* and/or *CDKN2A* mutations in the context of MAPK/PKA pathway alterations, a secondary analysis was performed focusing on their association with advanced neoplasia (Fig. 5). The inclusion of *CTNNB1* and/or *CDKN2A* mutations as markers of advanced neoplasia increased the sensitivity (by 2.1–7.2%), NPV (by 1.1–4.2%), and accuracy (by 0.4–1.6%) of PancreaSeq GC, but at the cost of reduced specificity (by 0.7–2.9%) and PPV (by 1.0–2.8%). According to McNemar’s test, only an improvement in sensitivity (*p* = 0.016) with the addition of both *CTNNB1* and *CDKN2A* reached statistical significance (Supplementary Fig. 5).

## Discussion

In this multi-institutional analysis, PancreaSeq GC demonstrated high diagnostic performance for pancreatic cyst classification and risk stratification. Among 241 EUS-FNA specimens, PancreaSeq GC achieved 95.0% accuracy for mucinous cysts and 93.4% accuracy for advanced neoplasia. It identified a broad spectrum of DNA and RNA alterations, including MAPK/PKA pathway mutations (*KRAS*, *GNAS*, and *BRAF*), tumor suppressor genes (*TP53*, *SMAD4*, *VHL*, and others), and gene fusion transcripts (*PRKACA/B*, *NTRK3*, and *BRAF*). Notably, *CEACAM5* overexpression was highly sensitive for mucinous cysts, while *CHGA* expression was associated with cPanNETs. Compared with traditional modalities, PancreaSeq GC showed statistically significant improvements in sensitivity, PPV, and overall accuracy for nonmucinous cysts, mucinous cysts, and advanced neoplasia.

An important aspect of this study is the enhanced accuracy of PancreaSeq GC compared with its predecessor, PancreaSeq, particularly in the detection of mucinous cysts and advanced neoplasia. This improvement can be attributed to PancreaSeq GC’s expanded genomic targets, which encompass a broad range of genomic alterations beyond a 22-gene DNA-based NGS panel. Specifically, PancreaSeq GC not only incorporates DNA point mutations and indels, but also chromosomal alterations, gene expression changes, and fusion gene transcripts. For instance, while *KRAS* and *GNAS* mutations are specific for mucinous cysts, there are potential challenges with mutant DNA assessment, such as in the setting of contaminating gastrointestinal tract contents. The inclusion of *CEACAM5* expression analysis in conjunction with *KRT7*/*KRT20* improves the inherent performance of PancreaSeq by increasing the sensitivity of detecting mucinous cysts. Since *CEACAM5* is the RNA correlate of CEA protein, this approach may eliminate the need to send a separate specimen for CEA testing, efficiently using limited amounts of pancreatic cyst fluid. In fact, elevated *CEACAM5* was more sensitive than CEA in the detection of mucinous cysts. Similarly, the addition of fusion genes increases the accuracy of PancreaSeq GC in identifying both mucinous cysts and advanced neoplasia. We previously described *BRAF* fusions as alternative MAPK driver genes in surgically resected *KRAS* wild-type IPMNs, which were missed preoperatively using PancreaSeq.^9^ PancreaSeq GC detected *BRAF* and *NTRK3* fusion genes in preoperative EUS-FNA specimens from *KRAS* wild-type IPMNs, highlighting the presence of multiple driver genes associated with the MAPK pathway.^23^ The inclusion of *PRKACA* and *PRKACB* fusion gene detection in PancreaSeq GC also plays an important role in the identification of mucinous cysts and advanced neoplasia.^16^ *PRKACA/B* fusion genes are defining genomic alterations for IOPNs, which are mucinous cysts that universally harbor advanced neoplasia. Thus, these RNA-based features enhance the diagnostic accuracy of PancreaSeq GC by providing orthogonal molecular evidence that complements established DNA-based mutational profiles associated with mucinous cysts and advanced neoplasia.

Another advancement of PancreaSeq GC over conventional molecular approaches to pancreatic cyst fluid testing is the ability to detect chromosomal alterations. Herein, we identified chromosomal alterations in 27% of specimens, which predominantly occurred in mucinous cysts and cPanNETs. Among mucinous cysts, these alterations were only identified in cases harboring advanced neoplasia. Springer et al*.* similarly reported a strong correlation between chromosomal alterations and the presence of advanced neoplasia.^24,25^ The authors found that 36% of mucinous cysts with advanced neoplasia exhibited losses of chromosomal arms in comparison with 4% of mucinous cysts with low-grade dysplasia. Chromosomal alterations have also been well-characterized in solid pancreatic neuroendocrine tumors (cPanNETs) and associated with an increased risk of distant metastasis.^9^ Pea et al*.* published solid PanNETs (sPanNETs) with multiple chromosomal alterations that had a 73% metastatic rate, which strongly correlated with the presence of alternative lengthening of telomere, a telomerase-independent telomere maintenance mechanism associated with aggressive tumor behavior.^26,27^ Lawrence et al*.* also identified recurrent chromosomal abnormalities, particularly in chromosome 10, as a risk factor for metastatic spread.^28^ Consistent with these findings, we found that ≥ 3 chromosomal alterations in cPanNETs had 100% sensitivity for the development of distant metastasis, although the specificity was limited to 64.3%. However, LOH at chromosome 10q was the most significant predictor of metastatic disease, achieving 100% sensitivity and 89.3% specificity. This chromosomal alteration outperformed traditional size criteria (> 2.0 cm), which showed 66.7% and 64.3% specificity for predicting distant metastasis. Of note, World Health Organization (WHO) grading remains the gold standard for PanNET risk stratification, but the limited cellularity of cytopathologic specimens from cPanNETs precludes accurate grading in most cases. Within this study, 16.7% of neuroendocrine-type neoplasms were diagnosed on the basis of cytopathologic evaluation alone. By integrating chromosomal alterations into a multiomic framework, PancreaSeq GC not only refines diagnostic precision for advanced neoplasia in mucinous cysts but also offers prognostic insights for metastatic risk in cPanNETs, underscoring its transformative potential in clinical practice.

In addition to *TP53* and *SMAD4*, we investigated the clinical relevance of detecting *CTNNB1* and *CDKN2A* alterations in mucinous cysts. Activating mutations in exon 3 of *CTNNB1*, which confer dysregulated Wnt/b-catenin pathway signaling, have been documented in IPMNs, but these alterations are rare, with a prevalence of only 4% as reported by Amato et al*.*^29^ In our analysis, 3 of 164 (2%) IPMNs harbored *CTNNB1* alterations, and 1 of 3 (33%) had high-grade dysplasia. By contrast, *CDKN2A* alterations were more frequently observed in mucinous cysts and associated with advanced neoplasia. *CDKN2A*, a tumor suppressor gene regulating the G1-S cell cycle checkpoint, was altered in 32 of 164 (20%) IPMNs and 1 of 18 (6%) MCNs. Moreover, 32 of 33 (97%) mucinous cysts had high-grade dysplasia and/or microscopic PDAC. Consistent with prior reports, *CDKN2A* alterations co-occurred with high-risk genomic alterations, such as *TP53*, *SMAD4*, *PIK3CA*, and *PTEN*, suggesting its involvement as a late genomic event in the malignant progression of IPMNs and MCNs.^30,31^ When incorporated as a marker of elevated risk in PancreaSeq GC, the presence of *CDKN2A* and/or *CTNNB1* mutations in combination with MAPK/PKA pathway alterations improved the sensitivity for detecting advanced neoplasia. Although this strategy resulted in marginal decreases in specificity and PPV, these results underscore the diagnostic utility of integrating infrequent yet diagnostically informative genomic events to refine risk stratification for advanced neoplasia in mucinous cysts.

Despite the significant findings, this study has several limitations that merit consideration. Although it utilized a multi-institutional cohort of prospectively collected EUS-FNA specimens, PancreaSeq GC testing was performed in a retrospective manner and only on surgically confirmed patient cases. This inherently introduces a selection bias toward clinically high-risk lesions that underwent surgical resection. While PancreaSeq GC demonstrated statistically significant performance over individual traditional markers for mucinous cysts, McNemar’s test showed no statistically significant difference between PancreaSeq GC and the combination of elevated fluid viscosity and elevated CEA. However, for approximately one-third of cases, fluid viscosity was not commented on within the EUS report, or the specimen was insufficient for CEA testing, limiting the interpretability of this comparison. Further, our comparison of diagnostic modalities did not include cyst fluid glucose and needle-based confocal laser endomicroscopy (nCLE), both of which are promising methods of pancreatic cyst evaluation, as these data were not collected in the original prospective cohort from which our samples were derived. Future studies should aim to compare PancreaSeq GC against a combination of established and emerging biomarkers. Nevertheless, PancreaSeq GC demonstrated higher performance as compared with traditional modalities and its predecessor, PancreaSeq. In addition, several chromosomal alterations associated with advanced neoplasia and distant metastatic disease for cPanNETs were identified, but the specific genes involved in these alterations require further investigation to fully understand their role in tumor progression. Although the inclusion of *CTNNB1* and *CDKN2A* modestly improved sensitivity for advanced neoplasia, it reduced specificity, highlighting the challenge of integrating lower-penetrance markers. Finally, this study did not evaluate the significance of low-level alterations, such as those previously described in *TP53* and *PIK3CA* as detected using PancreaSeq.

In summary, the PancreaSeq GC platform is a highly accurate multiomic tool for the classification of pancreatic cysts and the detection of advanced neoplasia from EUS-FNA specimens. The platform’s expanded genomic profiling, which includes a comprehensive analysis of DNA and RNA alterations, significantly improved diagnostic performance compared with both traditional methods and its predecessor, PancreaSeq. Notably, PancreaSeq GC demonstrated the ability to distinguish mucinous from nonmucinous cysts with high accuracy and effectively identify advanced neoplasia. Furthermore, PancreaSeq GC offers prognostic insights for cystic PanNETs by identifying multiple chromosomal alterations, such as LOH at chromosome 10q, which are predictive of metastatic risk. Although the study has limitations, particularly its focus on a surgically resected cohort, these findings support the clinical utility of PancreaSeq GC in refining diagnostic precision and guiding personalized management strategies for patients with pancreatic cysts.

## Supplementary Information

Below is the link to the electronic supplementary material.

Supplementary file1 (DOCX 44 KB)

Supplementary file2 (DOCX 17 KB)

Supplementary file3 (XLSX 40 KB)

Supplementary file4 (DOCX 1,177 KB)
