# Supplementary material for: PancreaSeq Genomic Classifier (PancreaSeq GC) Improves Pancreatic Cyst Classification and Detection of Advanced Neoplasia: A Multi-institutional Validation Study
Source: Ann Surg Oncol. 2025 Dec 12;33(4):2865–75. doi: 10.1245/s10434-025-18848-8 (PMC12982251; doi:10.1245/s10434-025-18848-8)
Supplement: Supplementary file 1 — Supplementary file1 (DOCX 44 KB) [file 10434_2025_18848_MOESM1_ESM.docx]

**Supplementary Materials and Methods**

*PancreaSeq specimen collection from Paniccia et al.^1^*

Upon EUS-FNA, pancreatic cyst fluid specimens are collected into a vial containing 600 uL of a DNA/RNA preservation solution. This nucleic acid preservation solution enables stabilization and prevents degradation by inactivation of pancreatic and gastrointestinal tract enzymes. The minimum specimen requirement is at least 200 uL of aspirated pancreatic cyst fluid. After collection, the vial is securely closed and inverted to mix the specimen with the preservation solution. The specimen is then transported frozen to the UPMC MGP laboratory for further processing, sequencing, and data analysis.

*PancreaSeq GC gene list*

The PancreaSeq Genomic Classifier interrogates genomic regions associated with pancreatic cysts to include single nucleotide variants and small insertions/deletions involving *AKT1*, *APC*, *BRAF*, *CTNNB1*, *GNAS*, *HRAS*, *IDH1*, *IDH2*, *KRAS*, *MEN1*, *MET*, *NF2*, *NRAS*, *PIK3CA*, *PTEN*, *STK11*, *TERT*, *TP53*, *TSC2*, and *VHL;* loss of heterozygosity (LOH) in in *RNF43* (17q), *SMAD4* (18q), *TP53* (17p), *VHL* (3p), *NF2* (22q), and *PTEN* (10q) tumor suppressors and copy number alterations (CNAs) at 13 chromosomal regions; gene fusions in *ALK* (partnering with *CCDC149, EML4, GFPT1*, *GTF2IRD1*, *STRN, TFG*), *BRAF* (partnering with *AGK*, *AKAP9*, *BCL2L11*, *CCNY*, *FAM114A2*, *GORASP2*, *MACF1*, *MKRN1*, *PICALM*, *POR*, *SND1*, *TRIM24*, *ZBTB8A* or *ZC3HAV1*), *ERBB4* (partnering with *EZR*), *NTRK1* (partnering with *BANP*, *ETV6, IRF2BP2*, *SQSTM1*, *SSBP2*, *TFG*, *TPM3*, or *TPR*), *NTRK3* (partnering with *EML4*, *ETV6*, *RBPMS, or SQSTM1*), *ROS1* (partnering with *CCDC30*), *RAF1* (partnering with *AGGF1*), *PRKACA* (partnering with *ATP1B1* or *DNAJB1*), and *PRKACB* (partnering with *ATP1B1*); and gene expression of *KRT7*, *KRT20*, *CHGA*, and *PGK1*.

*CEACAM5* mRNA expression by RT-qPCR

Primers and probes for the *CEACAM5* gene and the *GUSB* housekeeping control gene were designed and validated in-house to measure mRNA expression of these genes. One-step quantitative reverse transcription PCR (RT-qPCR) was performed using TaqMan One-step RT to Ct Master Mix kit and run on the ABI7500 real-time PCR instrument using the following parameters: 30 min at 48°C, 10 min 95°C, and 45 cycles of 15 seconds 95°C and 1 min at 60°C (catalog # 4392938, Applied Biosystems, Waltham, MA). Relative quantity of *CEACAM5* in Gene Expression Units (GEU) was calculated for each sample using the Ct difference between *CEACAM5* and *GUSB* compared to the same Ct difference calculated for normal pancreas RNA according to the2^-ΔΔCt^ method.^2^

*PancreaSeq GC data analysis*

A genomic classifier was developed to determine presence of a cystic precursor neoplasm, as defined as an IPMN, MCN, ITPN and IOPN, and to predict a presence of high-grade dysplasia and/or PDAC (advanced neoplasia) using in-house developed bioinformatic pipeline. For the prediction of a cystic precursor neoplasm, each detected genomic alteration was annotated to receive a value (0 to 3) based on the strength of its association with a cystic precursor neoplasm that included *KRAS (MAPK alterations)*, *GNAS*, *RNF43*, *BRAF*, *PRKACA*, and *PRKACB* alterations, *CEACAM5* mRNA expression, and mRNA expression of cytokeratin 7 (*KRT7*) and cytokeratin 20 (*KRT20*). A Genomic Classifier (GC) for cystic precursor neoplasms was calculated as a sum of the following individual values: GC cystic precursor neoplasm score = *x^KRAS^*^,^ *^GNAS^*^,^ *^RNF43^*^,^ *^BRAF^*^,^ *^PRKACA^* ^&^ *^PRKACB^* + *x^CEACAM5^* + *x^KRT7^*^/20^ + *x^CHGA^*, *x*=weighted value, 0 - 3. In parallel, *CHGA* expression is performed for each sample and evaluated for presence of a neuroendocrine tumor in samples that did not meet a cutoff for a cystic precursor neoplasm. A *CHGA* value of >50% was deemed a positive result. The prediction of advanced neoplasia arising from a cystic precursor neoplasm, a similar GC score was developed with each identified genomic alteration received a value (0 to 3) based on its association with high-grade dysplasia and PDAC. The values were derived from (1) an extensive literature and searchable database review to include but not limited to: TCGA, cBioPortal, and COSMIC, (2) an in-house database of >1000 pancreatic cyst fluid specimens with known surgical outcome, and postoperative surgical pancreatic specimens, and (3) RNA-Seq analysis of a subset of the aforementioned pancreatic cyst fluid and surgical specimens.^3-11^ Computational analysis of the molecular findings established a PancreaSeq GC for advanced neoplasia (risk score) that was calculated as a sum of the following individual values: GC risk score = *x*^SNV/indel^ + *x*^AF^ + *x*^CNA/LOH^ + *x*^GF^ + *x*^GEA^); *x*=weighted value, 0 - 3; SNV/indel (single nucleotide variant/small insertions and deletions, AF [allele frequency], CNA/LOH [copy number alteration/loss of heterozygosity], GF [gene fusions], and GEA [gene expression alterations] are indicators of genomic alteration type).

*Statistical analysis.*

Analyses were conducted on a case-level dataset in which the reference outcomes for mucinous cysts and advanced neoplasia, along with all candidate diagnostic indicators, were harmonized to binary factors (Absent, Present) through a standardized preprocessing routine. This included trimming whitespace, mapping common synonyms (e.g., “positive/pos/1/true” and “negative/neg/0/false,” including pancreas spelling variants) to canonical levels, and creating numeric derivations (1 for Present, 0 for Absent) for modeling. Receiver operating characteristic (ROC) curves were constructed for each feature–outcome pairing using the pROC package in R, and the area under the curve (AUC) was computed as a global measure of discrimination. Threshold selection for descriptive reporting employed Youden’s J index (sensitivity + specificity − 1), identifying the operating point that maximized the joint trade-off between true-positive and true-negative rates. Instances with insufficient variability (e.g., a single class) were detected and excluded from ROC computation, with limitations noted. For visualization-only edge cases that produced degenerate coordinate sets, plotted coordinates were augmented to include (0,0) and/or (1,1) to preserve interpretability, while AUC and p-values remained based on the original ROC object. Feature labels for display matched their clinical descriptors (e.g., “Increased Viscosity,” “Elevated CEA,” “PancreaSeq”), whereas all statistical calculations operated on binary encodings.

*Pairwise comparative analysis (McNemar framework).*

To compare diagnostic performance between tests, a paired design based on McNemar’s test was implemented across five metrics—sensitivity, specificity, positive predictive value (PPV), negative predictive value (NPV), and overall accuracy—separately for mucinous cysts and advanced neoplasia. For each metric and test pair, the analysis constructed the appropriate subset: for sensitivity, only reference-positive cases were considered; for specificity, only reference-negative cases; for PPV, comparisons were based on cases where at least one test was positive; for NPV, the analogous approach was applied to negative calls; and for accuracy, correctness was assessed across all cases. After restricting to the metric-specific subset, 2×2 paired contingency tables were built on correctness (or metric-appropriate success), and discordant pairs were counted. If no discordant pairs were present, the tests were considered equivalent (p = 1). Exact p-values were obtained using the exact2x2 package in R, with fallback to the asymptotic McNemar test from the stats package when necessary. To avoid spurious inference from sparse data, the pipeline required at least 10 valid observations in the relevant subset (e.g., ≥10 reference positives for sensitivity) and flagged cases with insufficient data or no variation. This approach produced a complete matrix of pairwise p-values for all tests and metrics, enabling a rigorous assessment of where performance differences were statistically significant and where differences were either unsupported or not evaluable.

*Visualization, software, and reproducibility.*

ROC figures were generated using ggplot2 with a minimalist theme to optimize clarity, with sensitivity on the y-axis, 1 – specificity on the x-axis, an equal aspect ratio, and a gray diagonal reference line denoting random classification. Youden-optimal operating points were highlighted with red markers. Single-feature ROC panels were exported at high resolution for each feature against both reference outcomes, and a composite two-row panel summarized the two result families in a unified layout. For the paired comparisons, upper-triangle heatmaps of McNemar p-values were created for each metric and condition using ggplot2, encoding significance thresholds (<0.001, <0.01, <0.05) with a sequential color palette, “NS” for non-significant results, “Identical” for zero discordant pairs, and “No Data” when validity checks failed. Each tile displayed either the formatted p-value or an informative issue tag (e.g., “Insuff. TP/TN,” “No Pos/Neg,” “No Variation”), and long test names were wrapped for readability. Heatmaps for all five metrics were arranged in a five-column grid for each condition, with mucinous cysts and advanced neoplasia displayed in separate rows, and the combined figure was exported at publication-quality resolution. All analyses were performed in R (version ≥4.0) using the following packages: pROC for ROC analysis, exact2x2 for exact McNemar tests, ggplot2 for visualization, dplyr for data manipulation, caret for preprocessing utilities, and gridExtra, gtable, and grid for figure assembly. This combination of tools ensures reproducibility and transparency of the workflow.

**Supplementary References**

1. Paniccia A, Polanco PM, Boone BA, et al. Prospective, Multi-Institutional, Real-Time Next-Generation Sequencing of Pancreatic Cyst Fluid Reveals Diverse Genomic Alterations that Improve the Clinical Management of Pancreatic Cysts. Gastroenterology 2022.

2. Livak KJ, Schmittgen TD. Analysis of relative gene expression data using real-time quantitative PCR and the 2(-Delta Delta C(T)) Method. Methods 2001;25:402-8.

3. Cancer Genome Atlas Research Network. Electronic address aadhe, Cancer Genome Atlas Research N. Integrated Genomic Characterization of Pancreatic Ductal Adenocarcinoma. Cancer Cell 2017;32:185-203 e13.

4. Cerami E, Gao J, Dogrusoz U, et al. The cBio cancer genomics portal: an open platform for exploring multidimensional cancer genomics data. Cancer Discov 2012;2:401-4.

5. Tate JG, Bamford S, Jubb HC, et al. COSMIC: the Catalogue Of Somatic Mutations In Cancer. Nucleic Acids Res 2019;47:D941-D947.

6. Singhi AD, Zeh HJ, Brand RE, et al. American Gastroenterological Association guidelines are inaccurate in detecting pancreatic cysts with advanced neoplasia: a clinicopathologic study of 225 patients with supporting molecular data. Gastrointest Endosc 2016;83:1107-1117 e2.

7. Singhi AD, Nikiforova MN, Fasanella KE, et al. Preoperative GNAS and KRAS testing in the diagnosis of pancreatic mucinous cysts. Clin Cancer Res 2014;20:4381-9.

8. Singhi AD, McGrath K, Brand RE, et al. Preoperative next-generation sequencing of pancreatic cyst fluid is highly accurate in cyst classification and detection of advanced neoplasia. Gut 2018;67:2131-2141.

9. Nikiforova MN, Khalid A, Fasanella KE, et al. Integration of KRAS testing in the diagnosis of pancreatic cystic lesions: a clinical experience of 618 pancreatic cysts. Mod Pathol 2013;26:1478-87.

10. Singhi AD, Wood LD, Parks E, et al. Recurrent Rearrangements in PRKACA and PRKACB in Intraductal Oncocytic Papillary Neoplasms of the Pancreas and Bile Duct. Gastroenterology 2020;158:573-582 e2.

11. Singhi AD, George B, Greenbowe JR, et al. Real-Time Targeted Genome Profile Analysis of Pancreatic Ductal Adenocarcinomas Identifies Genetic Alterations That Might Be Targeted With Existing Drugs or Used as Biomarkers. Gastroenterology 2019;156:2242-2253 e4.
