# Supplementary material for: PancreaSeq Genomic Classifier (PancreaSeq GC) Improves Pancreatic Cyst Classification and Detection of Advanced Neoplasia: A Multi-institutional Validation Study
Source: Ann Surg Oncol. 2025 Dec 12;33(4):2865–75. doi: 10.1245/s10434-025-18848-8 (PMC12982251; doi:10.1245/s10434-025-18848-8)
Supplement: Supplementary file 2 — Supplementary file2 (DOCX 17 KB) [file 10434_2025_18848_MOESM2_ESM.docx]

**Supplementary Data**

*Patient demographics and cyst characteristics*

This study cohort comprised 241 patients who underwent endoscopic ultrasound-guided fine-needle aspiration (EUS-FNA) of pancreatic cyst fluid specimens with corresponding clinicopathologic data and PancreaSeq Genomic Classifier testing. The cohort included 131 female patients (54.4%) and 110 male patients (45.6%), with ages ranging from 25 to 91 years (mean age: 65.8 years, median age: 68 years). The majority of cysts were located in the head, uncinate process, or neck of the pancreas (n=131, 54.4%), while the remainder were found in the body or tail (n=110, 45.6%). All cysts included in this study were clinically determined to be in the side-branch or peripheral location of the pancreas and were not centered within the main pancreatic duct, representing lesions amenable to EUS-FNA sampling. Pancreatic cyst sizes varied considerably across the cohort, ranging from 1.0 cm to 15.0 cm in maximum dimension where documented (mean size: 3.5 cm, median size: 3.0 cm). Cyst fluid viscosity was assessed in 235 cases, with viscous fluid observed in 166 cases (70.6%) and nonviscous fluid in 69 cases (29.4%). Six cases had no viscosity data available.

*Clinical presentation and imaging features*

Clinical symptoms were documented in 241 patients, with 85 patients (35.3%) presenting with associated symptoms and 156 patients (64.7%) being asymptomatic. Among the symptomatic patients, jaundice was specifically evaluated and found to be present in 29 patients (12.0% of total cohort) where this information was documented. The presence of increasing cyst size on follow-up imaging was assessed in a subset of patients, with 64 patients (26.6%) showing interval growth, 47 patients (19.5%) showing stable size, and the remainder having no available comparative imaging data.

Mild-to-significant ductal dilatation was present in 114 cases (47.7%) where this feature was evaluated. Mural nodules, an important imaging feature associated with higher-risk lesions, were identified in 64 cases (26.6% of the cohort). The combination of worrisome features including ductal dilatation, mural nodules, and increasing cyst size was variably present across the cohort, with many high-risk lesions demonstrating multiple concerning features.

*Laboratory and cytopathologic findings*

Carcinoembryonic antigen (CEA) levels in cyst fluid were measured in 179 cases, with elevated CEA (>192 ng/mL) documented in 120 cases (67.0% of tested cases) and non-elevated CEA in 59 cases (33.0% of tested cases). CEA data were not available for 62 cases (25.7% of total cohort). Cytopathologic examination was performed on all specimens, with malignant cytology identified in 49 cases (20.3% of the cohort). However, it is notable that malignant cytology was absent in the majority of cases (n=192, 79.7%), highlighting the complementary role of molecular testing in risk stratification, as many cases with negative or indeterminate cytology harbored high-risk genomic alterations.

*Molecular (PancreaSeq and PancreaSeq GC) testing*

PancreaSeq testing identified pathogenic alterations in 210 cases (87.1%), while 31 cases (12.9%) had negative molecular results. The PancreaSeq Genomic Classifier, which incorporates additional copy number alterations (CNAs) and fusion analysis, detected abnormalities in 217 cases (90.0%), demonstrating enhanced sensitivity compared to standard mutation analysis alone. Among the most common alterations, *KRAS* mutations were identified in 168 cases, *GNAS* mutations in 103 cases, *TP53* alterations in 78 cases, and *SMAD4* alterations in 62 cases, with mutation frequencies varying significantly by diagnosis type and reflecting the underlying biology of different cystic neoplasm subtypes. *CEACAM5* expression levels were evaluated in 228 cases, with elevated levels detected in 175 cases (76.8%) and non-elevated levels in 53 cases (23.2%). *CEACAM5* data were not available for 13 cases. Chromogranin A (*CHGA*) expression was assessed to identify neuroendocrine differentiation, with elevated *CHGA* found in 40 cases (16.6% of the cohort) and non-elevated *CHGA* in 201 cases (83.4%).

*Surgical pathology follow-up*

The final histopathologic diagnoses encompassed a wide spectrum of pancreatic cystic neoplasms. The largest diagnostic category was intraductal papillary mucinous neoplasm (IPMN) with low-grade dysplasia (LGD), comprising 77 cases (32.0% of the cohort). IPMN-associated pancreatic ductal adenocarcinoma (PDAC) represented 66 cases (27.4%), constituting the most common malignant diagnosis. IPMN with high-grade dysplasia (HGD) was diagnosed in 21 cases (8.7%). Cystic pancreatic neuroendocrine tumors (PanNETs) comprised 37 cases (15.4% of the cohort), representing the second most common diagnostic category. Mucinous cystic neoplasms (MCNs) accounted for 16 cases total, including 4 cases of MCN-associated PDAC (1.7%), 2 cases of MCN with HGD (0.8%), and 10 cases of MCN with LGD (4.1%). Less common diagnoses included serous cystadenomas (n=13, 5.4%), intraductal oncocytic papillary neoplasms (IOPNs, n=3, 1.2%), and rare entities such as solid-pseudopapillary neoplasm (n=2, 0.8%), intraductal tubulopapillary neoplasm (ITPN, n=1, 0.4%), acinar cell carcinoma (n=1, 0.4%), mixed serous cystadenoma-neuroendocrine neoplasm (n=1, 0.4%), paraganglioma (n=1, 0.4%), cystic schwannoma (n=1, 0.4%), and non-neoplastic cysts (n=2, 0.8%).
