# Supplementary figures and images for: PancreaSeq Genomic Classifier (PancreaSeq GC) Improves Pancreatic Cyst Classification and Detection of Advanced Neoplasia: A Multi-institutional Validation Study
Source: Ann Surg Oncol. 2025 Dec 12;33(4):2865–75. doi: 10.1245/s10434-025-18848-8 (PMC12982251; doi:10.1245/s10434-025-18848-8)

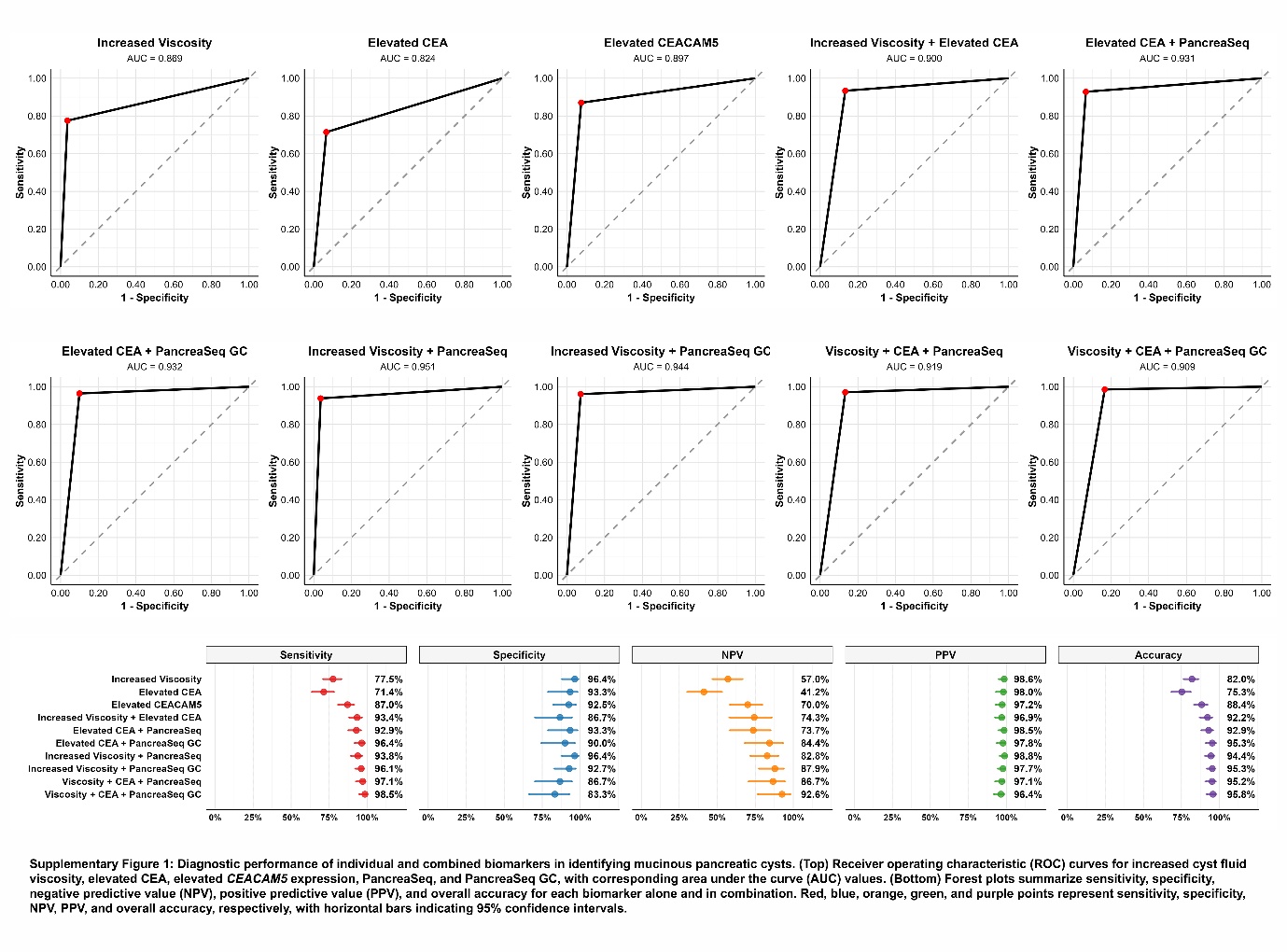


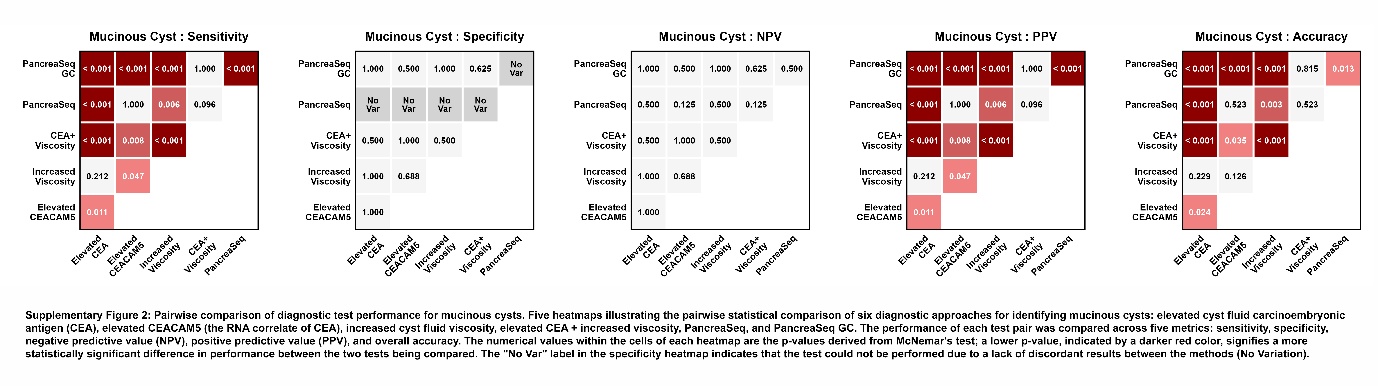


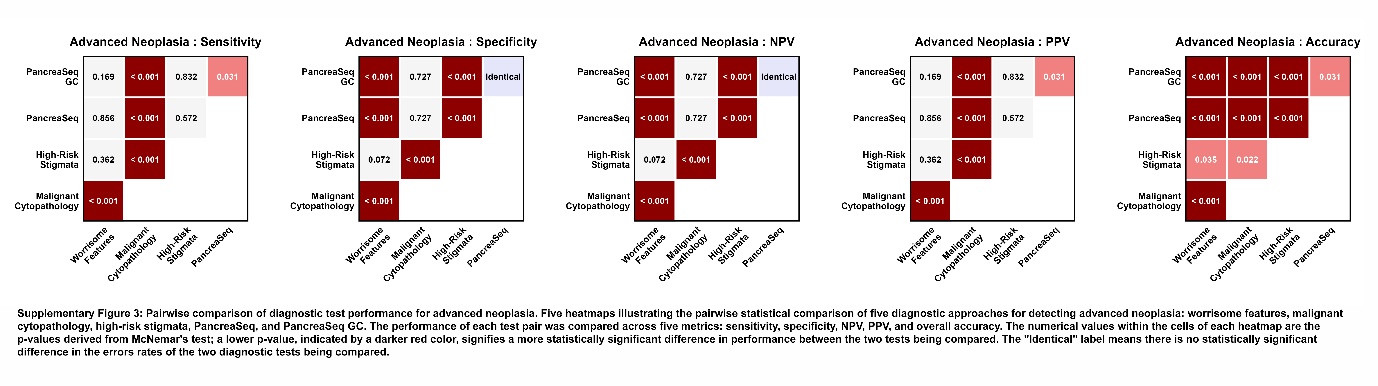


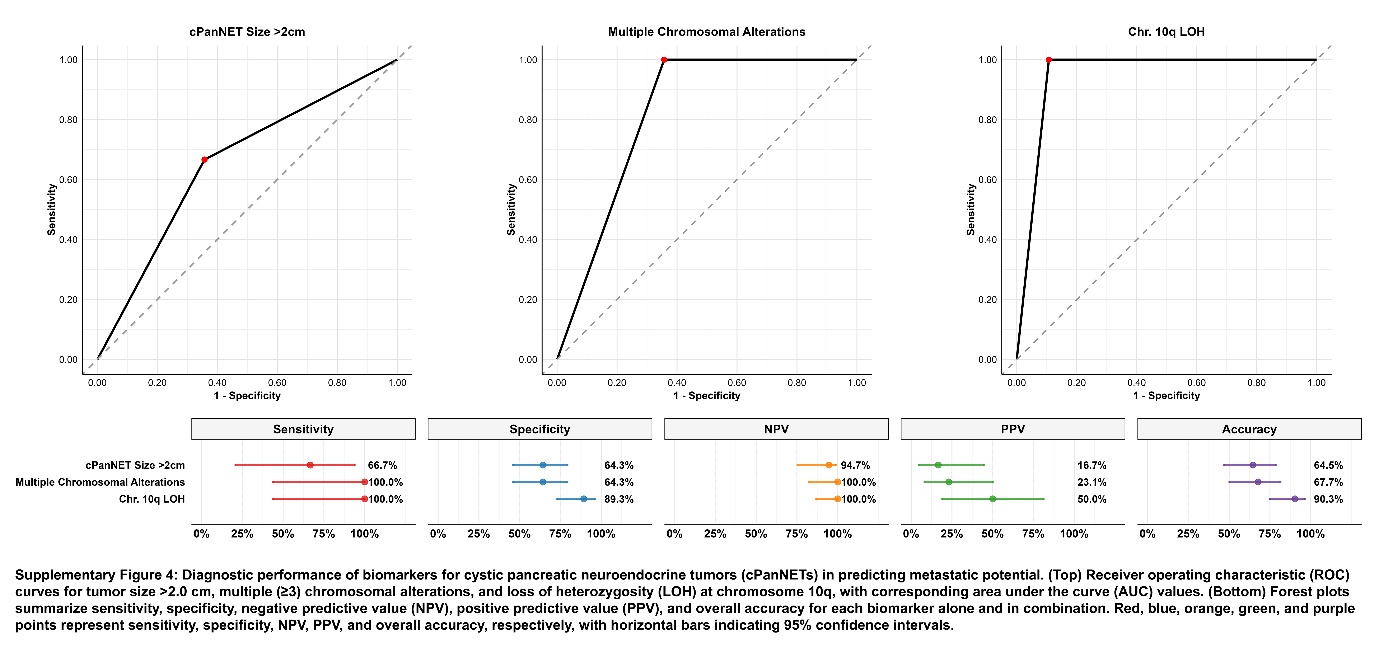


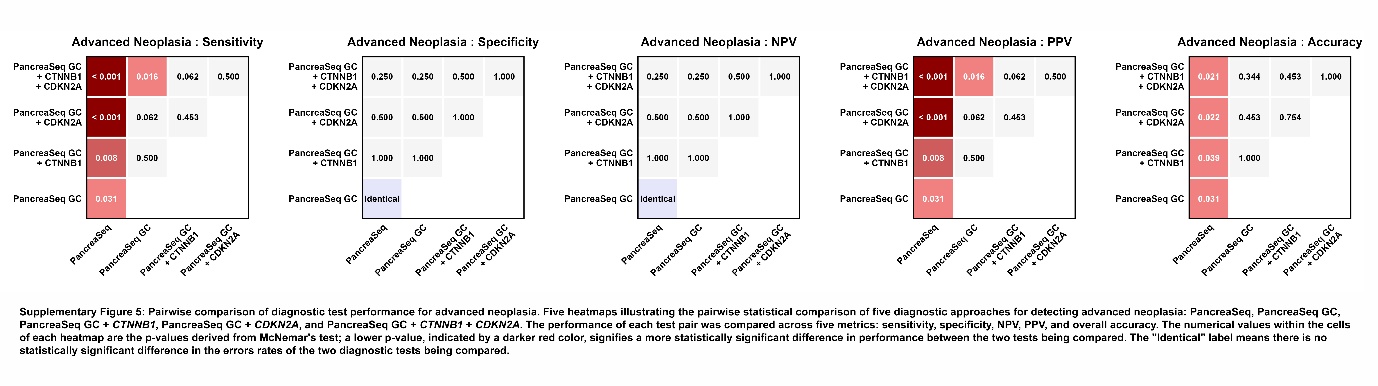

Supplement: Supplementary file 4 — Supplementary file4 (DOCX 1,177 KB) [file 10434_2025_18848_MOESM4_ESM.docx]
